# Oral administration of inactivated porcine epidemic diarrhea virus activate DCs in porcine Peyer’s patches

**DOI:** 10.1186/s12917-018-1568-z

**Published:** 2018-08-16

**Authors:** Chen Yuan, En Zhang, Lulu Huang, Jialu Wang, Qian Yang

**Affiliations:** MOE Joint International Research Laboratory of Animal Health and Food Safety, College of veterinary medicine, Nanjing Agricultural University, Weigang 1, Nanjing, Jiangsu 210095 People’s Republic of China

**Keywords:** Peyer’s patches, Dendritic cells, Inactivated porcine epidemic diarrhea virus

## Abstract

**Background:**

Peyer’s patches (PPs) can be considered as the immune site of the intestine. Within PPs, Dendritic cells (DCs) can uptake antigens from the gut lumen by extending dendrites into epithelium, and process it and then present to lymphocytes, which effectively antigen produces an immune response. Porcine epidemic diarrhea virus (PEDV) is the causative agent of porcine epidemic diarrhea (PED), an acute and highly contagious enteric viral disease. The interaction between inactivated porcine epidemic diarrhea virus and porcine monocyte-derived dendritic cells (Mo-DCs) has been reported. However, little is known about the interaction between inactivated PEDV and DCs in porcine PPs.

**Results:**

In this study, for the first time we investigated the role of DCs in porcine PPs after oral administration inactivated PEDV. Firstly, a method to isolate DCs from porcine PPs was established, in which the purity of SWC3a^+^/MHC-II^+^ DCs was more than 90%. Our findings clearly indicate that DCs in porcine PPs after oral administration of inactivated PEDV not only stimulated the proliferation of allogeneic lymphocytes, but also secreted cytokines (IL-1, IL-4). Furthermore, the number of DCs and IgA^+^ cells in porcine intestinal mucosal significantly increased and the levels of anti-PEDV specific IgG antibody in the serum and SIgA antibody in the feces increased after oral administration inactivated PEDV.

**Conclusions:**

Our findings indicate that oral administration of inactivated PEDV activate DCs in porcine Peyer’s patches and inactivated PEDV may be a useful and safe vaccine to trigger adaptive immunity.

## Background

Gut is the major immune organ of the body and the intestinal mucosa is thought to be the primary site for performing local-specific immune responses. Gut associated lymphoid tissue (GALT), consists of isolated or aggregated lymphoid follicles forming Peyer’s patches (PPs), is considered to be the key inductive tissues for the mucosal immune system [1]. PPs are known as the immune sensors of the intestine because of their ability to transport luminal antigens and bacteria into organized lymphoid tissues within the intestinal mucosa [2]. PPs contain too many immunocompetent cells that are required for the generation of an immune response. Dendritic cells (DCs) are professional antigen-presenting cells (APC) that possess the unique capacity to trigger primary adaptive immune responses through the antigen-specific activation of naive T cells. DCs in PPs can extend dendrites into the lumen to capture antigens and then present to resting T cells and thus initiate adaptive immune responses [3, 4].

According to the cell lineage, DCs can be divided into two major subsets that include plasmacytoid DCs (pDCs) and myeloid DCs, with the latter commonly referred to as conventional DCs (cDCs) [5]. DCs involved in intestinal immunity were investigated by cDCs induced from bone marrow cells or pDCs induced from blood mononuclear cells so far [6, 7]. But neither pDCs nor cDCs represent the reality of DCs within PPs. Indeed it is difficult to obtain the DCs within PPs in human. Unlike the mouse (isolated PPs), pig had continuous PPs (aggregated PPs), just like human. It is possible to get DCs within PPs in pig. The pig would be an animal model for studying DCs within PPs in human. Therefore, it is important to establish a method that isolate DCs from porcine PPs.

Porcine epidemic diarrhea virus (PEDV) is the causative agent of porcine epidemic diarrhea (PED), an acute and highly contagious enteric viral disease. PEDV infects epithelia in both small and large intestine and cause diarrhea, dehydration, and a high mortality in piglets [8–10]. Currently, PED is globally recognized as an emerging and reemerging disease that has resulted in great economic losses to the swine industry worldwide. PEDV infect piglets mainly through fecal-oral route (digestive tract). Vaccination is a potent tool in the control and prevention of PED [11]. It is important to develop oral vaccines that can elicit effective mucosal immune responses against PEDV infection. Oral vaccines could cut off the route of PEDV invasion. Oral administration of vaccine in pigs have been successfully used to prevent infectious disease [12, 13].

In our previous work, we reported that the interaction between inactivated PEDV and porcine Mo-DCs, which inactivated PEDV enhances the ability of DCs to present, migrate and induce the activation of T lymphocytes in vivo and in vitro [6]. However, little is known about the interaction between inactivated PEDV and DCs in porcine PPs. In this study, for the first time we investigated the role of DCs in porcine PPs after oral inoculation of inactivated PEDV. Firstly, a method to isolate DCs in porcine PPs was established, in which the purity of SWC3a+/MHC-II+ DCs was more than 90%. Our findings clearly indicated that PPs DCs from porcine intestinal mucosal after oral delivery of inactivated PEDV not only stimulated the proliferation of allogeneic lymphocytes, but also secreted cytokines (IL-1,IL-4). Furthermore,the number of DCs and IgA antibody levels in porcine intestinal mucosal significantly increased as compared with the control group and the levels of anti-PEDV specific IgG antibody in the serum and SIgA antibody in the feces increases after oral inactivated PEDV.

## Methods

### Experimental design

A total of 6 male (cross-bred Duroc/Landrace/Yorkshire) piglets: aged 2-day-old; weight, 1.30–1.50 kg, were obtained from Jiangsu Academy of Agricultural Sciences (Nanjing, China). The piglets were born via natural farrow and were fed with milk. The piglets were housed in Jiangsu Academy of Agricultural Sciences Pig Farm with a constant humidity (60%) and temperature (26 °C) at 12 h light/dark cycle. The PEDV (CV777) was propagated on Vero cells in DMEM (GIBCO, USA) with 5% fetal bovine serum and purified the collection by sucrose gradient centrifugation. Ultraviolet rays inactivated PEDV (UV-PEDV) were produced by exposing the virus to ultraviolet rays for 6 h at an optimal cross linking value. The piglets were randomly divided into two groups (3 piglets each group), respectively fed with UV-inactivated PEDV (100μg/dose) or equal volume of PBS twice at days 5 and 30 for two months. The next week after the last administration, these pigs were euthanized by intravenous injection of pentobarbital sodium (100 mg/kg) and piglets were sacrificed and ileum samples were collected. All procedures performed on the animals were approved by the Institutional Animal Care and Use Committee of Nanjing Agricultural University and followed the National Institutes of Health guidelines for the performance of animal experiments.

### Reagents

FITC-MHCII, PE-SWC3a were from Abcam (New Territories, Hong Kong). Dylight 488-, 594-,-conjugated secondary antibodies were purchased from MultiSciences (Lianke) Biotech Co., Ltd. (China). 4′, 6-diamidino-2-phenylindole (DAPI) solution were obtained from Jackson ImmunoResearch Laboratories (West Grove, PA, USA). CFSE (carboxyfluorescein succinimidyl amino ester) were purchased from Invitrogen (USA). The Cytokine test kits were purchased from Shanghai Huyu Biotechnology (Shanghai, China). Cell Counting Kit-8 were purchased from Beyotime Biotechnology (China).

### Isolation of DCs in porcine PPs

After removal of residual mesenteric fat tissue, the ileum was then cut into 1.5 cm pieces. The pieces were incubated in 20 ml of 5 mM EDTA in HBSS for 20 min at 4 °C. Then centrifuged and discarded the supernatant. The ileum was cut in 1 cm pieces and placed in digestion solution containing 4% fetal bovine serum, 2 mg/ml each of Collagenase D (Roche) and DNase I (Sigma), and 100 U/ml Dispase (Fisher) at 37 °C for 20 min with slow rotation. The supernatants were obtained by density gradient centrifugation and then sorted DCs marked SWC3a and MHC-IIfrom porcine PPs by fluorescence activated cell sorting (FACS).

### Autologous mixed lymphocyte reaction

DCs had the ability to stimulate lymphocyte proliferation. We examined whether DCs stimulated lymphocyte proliferation in two ways. Firstly, Different groups DCs were incubated with allogeneic lymphocyte labeled CFSE, at a rate of DCs: lymphocyte = 1: 10. Five days later, the proliferation of lymphocyte was detected by FACS.Another way, different groups DCs were incubated with allogeneic lymphocyte, five days later, the proliferation of lymphocyte was detected by CCK8 (Cell Counting Kit-8).

### Cytokine assays by enzyme-linked immunosorbent assay

Different groups DCs were incubated with allogeneic lymphocyte for 24 h, at a rate of DCs: lymphocyte = 1:10. The cytokines (IL-2, IL-4, IL-6 and IL-10) in culture mediums were measured using enzyme-linked immunosorbent assay and performed according to the manufacturer’s instructions.

### Immunofluorescence (IF) staining of DCs

Tissue sections were permeabilized in 0.4% Triton X-100 in PBS for 5 min. After treating with 5% bovine serum albumin in PBS for 1 h, the tissue sections were incubated with the SWC3a or MHC-IIprimary antibodies overnight at 4 °C, PBS was used in place of the anti-pig antibody for the control. After rinsing in PBS, sections incubated with Alexa Fluor 488 or 647 labeled secondary antibodies were kept at room temperature for 1 h. After staining with DAPI, the cryosections were observed under a confocal laser microscope (LSM-710; Zeiss, Oberkochen, Germany) visualized by CLSM (LSM 710, Zeiss, Oberkochen, Germany).

### Immunohistochemistry

After deparaffinization and rehydration, paraffin sections were put in citrate buffer (pH 6) at 90–95 °C for 15 min to retrieve antigen. Then, the sections were put in 0.3% H_2_O_2_ to quench endogenous peroxidase and washed in PBS. 5% bovine serum albumin were incubated on sections for 30 min to close the non-specific antibody binding sites. After blocking with 5% bovine serum albumin, sections were incubated with goat anti-pig IgA overnight at 4 °C. Biotinylated secondary antibodies were added to the sections for 1 h at room temperature, and treated with SABC for 60 min. Sections were counterstained with hematoxylin and images were obtained using a light microscope (BH-2; Olympus). Different fields of each tissue in each piglet were counted for the statistical analysis.

### Enzyme-linked immunosorbent assay (ELISA)

The PEDV-specific IgA in feces and PEDV-specific IgG in serum were detected by ELISA. ELISA plates were coated 2 μg purified PED*V*/*w*ell at 4 °C overnight. After antigen removal, ELISA plates were blocked with 3% bovine serum albumin (BSA) in PBS which contains 0.05% Tween (PBST) for 2 h at 37 °C. Then, 100-fold dilutions of serum samples or 2-fold dilutions of lavage fluid from pig were added to the plates and incubated at 37 °C for 2 h. Washed with PBST and added 100 μl of HRP-conjugated goat anti-pig IgA/IgG antibody at 1:2000 dilution and incubated at 37 °C for 1 h. Plates were washed 5 times and incubated with 3, 3′, 5, 5′-tetramethylbenzidine (TMB) for 15 min. Then, the reaction was stopped with sulfuric acid (2 M). Optical densities at 450 nm were measured using an enzyme-linked immunosorbent assay (ELISA) plate reader.

### Statistical analysis

Results were expressed as means ± SD. Analysis of variance and unpaired Student’s t-tests were employed to determine statistical differences among multiple groups. *P* values < 0.05 were considered significant (*P < 0.05, **P < 0.01).

## Results

### Sorting DCs from porcine PPs

**Fig. 1:**
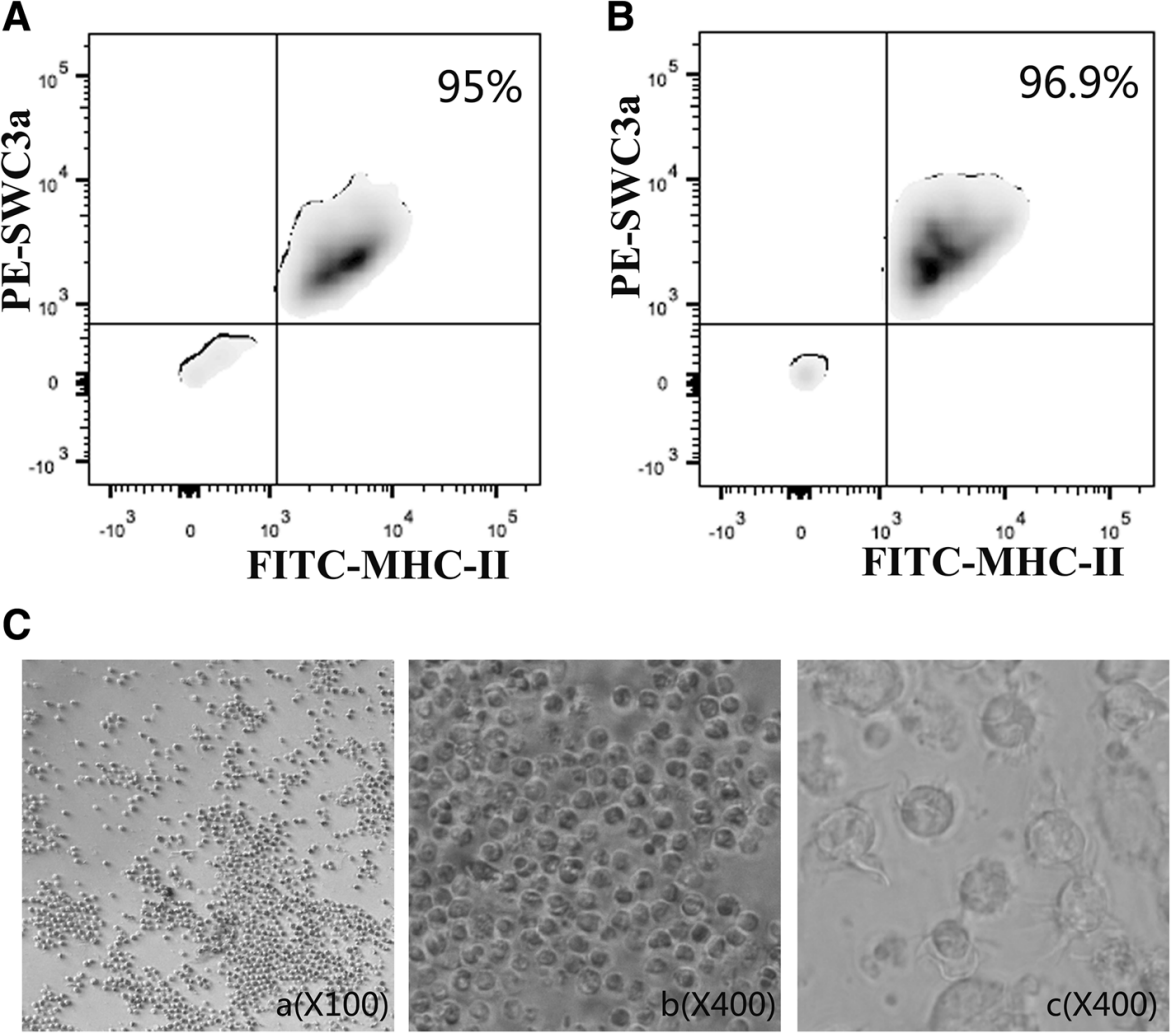
Sorting DCs from porcine PPSs. two days old piglets were given PBS or inactivated PEDV (100μg/dose) every week for two months. SWC3a^+^/MHC-II^+^ DCs from conventional healthy pigs (**a**) and oral inactivated PEDV (**b**) were sorted and analyzed by FACS., *n* = 6. **c** Morphology of DCs from porcine PPs under a light microscope

PPs are important inductive sites for the initiation of innate and adaptive immune responses [2]. To successfully isolate DCs from porcine PPs is an important task, which might contribute to further study on the role of DCs in porcine intestinal mucosal PPs. Cell suspensions were prepared in porcine PPs from conventional healthy pigs and oral inactivated PEDV for two months, and sorted by FACS according to DCs maker SWC3a and MHC-II. Then SWC3a^+^ /MHC-II^+^ DCs in porcine PPs were analyzed by FACS. The purity of SWC3a^+^/MHC-II^+^ DCs from conventional healthy pigs and oral inactivated PEDV for two months were 95% and 96.9% respectively (Fig. 1a, b). After 5 days of culture, there were many dendrite-like processes on the surface of the DCs from porcine Peyer’s patches under the inverted microscope (Fig. 1c).

### DCs can promoted the proliferation of allogeneic lymphocytes

**Fig. 2:**
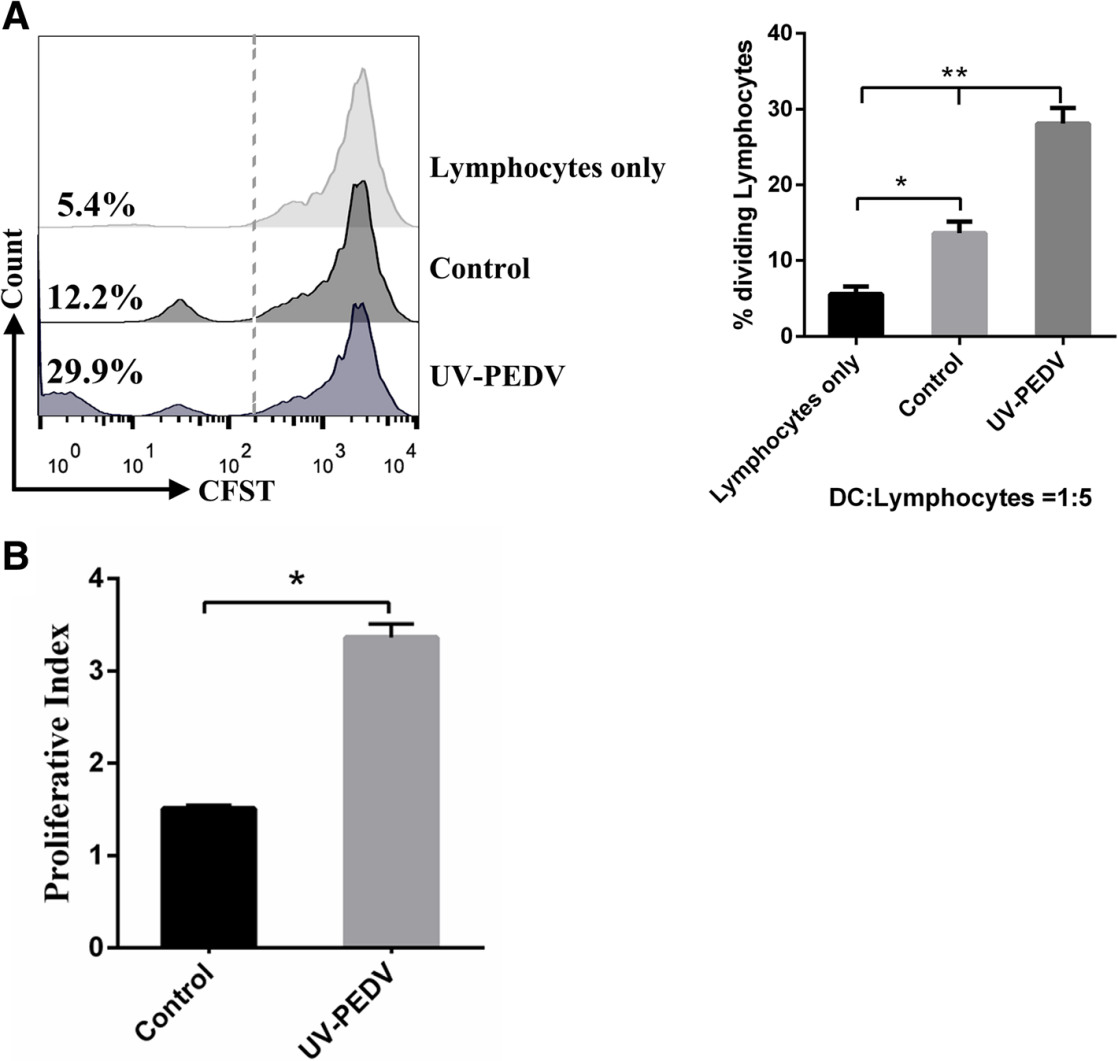
DCs can promoted the proliferation of allogeneic lymphocytes. **a** DCs in PPs from conventional healthy pigs and oral inactivated PEDV for two months co-cultured with allogeneic lymphocytes at a ratio of 1:10.lymphocytes proliferation was analyzed by CCK8. **b** DCs from intestinal mucosal PPs from conventional healthy pigs and oral inactivated PEDV for two months co-cultured with CFSE-labeled allogeneic lymphocytes at a ratio of 1:10.lymphocytes proliferation was analyzed by CFSE dilution using FACS. Percentages refer to proportion of lymphocytes that proliferated within 5 days. Data are represented as mean ± S.D. One representative of three similar independent experiments is shown

DCs can uptake antigens from the gut lumen by extending dendrites into epithelium, then process and present to lymphocytes, which effectively antigen produces an immune response [4, 14, 15]. So in next step, we investigated the potential of DCs to prime lymphocytes responses after its interactions with lymphocytes. As the result showed, DCs in porcine PPs from oral inactivated PEDV pigs for two months had a significantly increased ability to promote lymphocytes proliferation (Fig. 2a, b), while that of DCs from conventional healthy pigs had no significant changes (Fig. 2b).

### DCs can stimulate lymphocytes to secrete cytokines

**Fig. 3:**
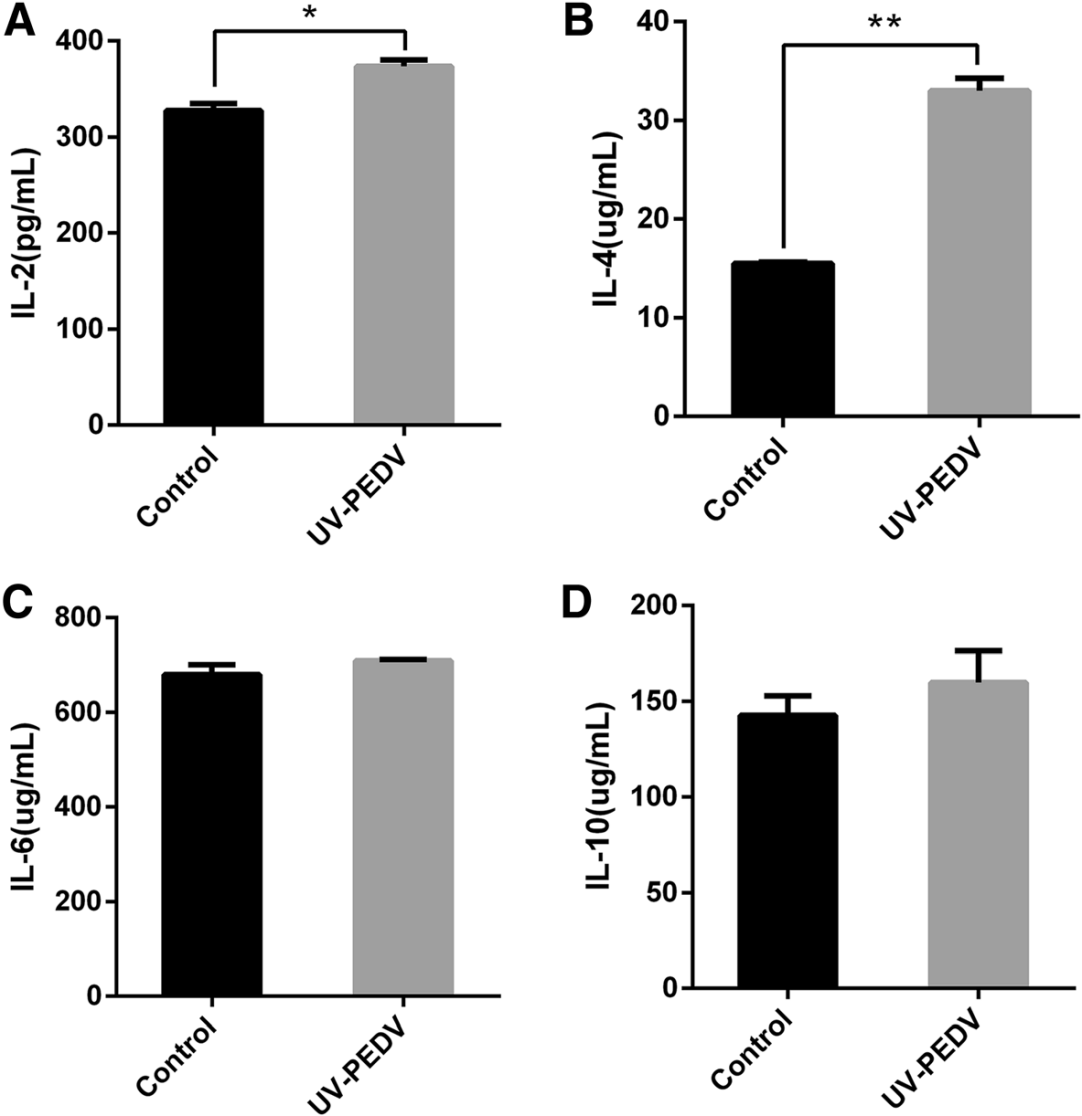
DCs can stimulate lymphocytes to secrete cytokines. DCs in porcine PPSs from conventional healthy pigs and oral inactivated PEDV for two months co-cultured with allogeneic lymphocytes at a ratio of 1:5 for 24 h respectively, the culture supernatants were collected. IL-2 (**a**), IL-4 (**b**), IL-6 (**c**) and IL-10 (**d**) release in culture supernatants were measured by ELISA. All of the data are presented as means ± SD of three replicates and are representative of three independent experiments. *P < 0.05; **P < 0.01

To examine the effect of DCs stimulate lymphocytes on cytokines, we measured the level of IL2, IL-4, IL-6 and IL-10 in DCs co-cultured with allogeneic lymphocytes by ELISA kit. As the result showed, DCs in porcine PPs from oral inactivated PEDV pigs for two months co-cultured with allogeneic lymphocytes significantly stimulated the secretion of IL2, IL-4 by lymphocytes, as compared with the control group (Fig. 3a, b). However, there was no statistical difference in level of IL-6 and IL-10 between the treated and control group in DCs co-cultured with allogeneic lymphocytes (Fig. 3c, d).

### The number of ileum DCs increases after oral inactivated PEDV

**Fig. 4:**
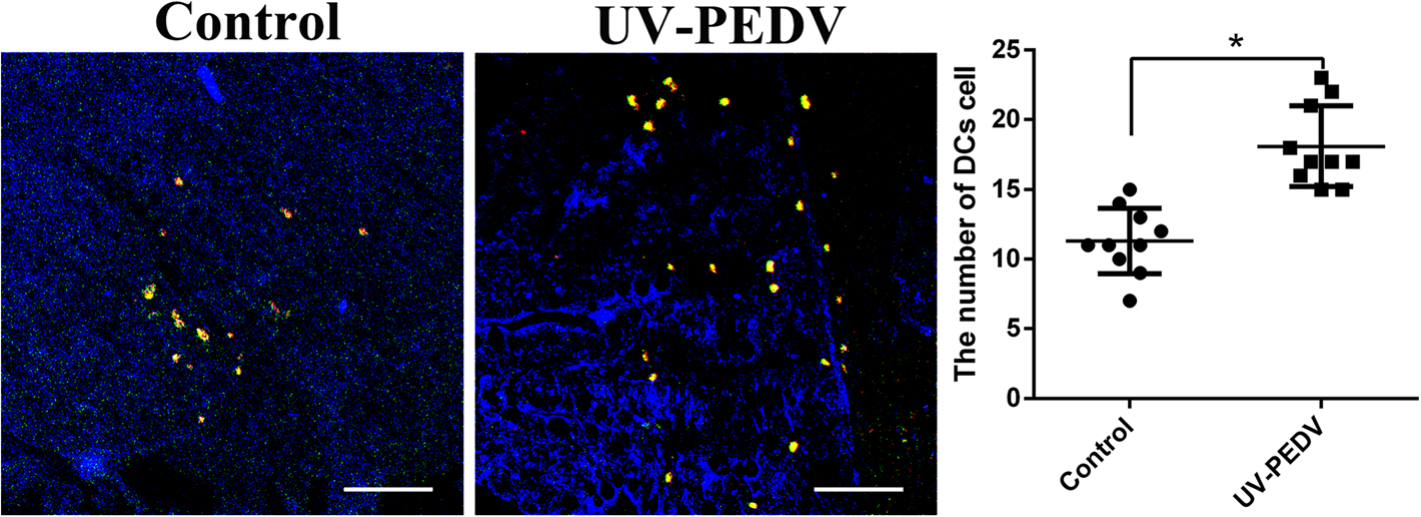
The number of ileum SWC3a^+^/MHC-II^+^DCs increases after oral inactivated PEDV. SWC3a^+^/MHC-II^+^ DCs were showed by Immunofluorescence staining. The number of SWC3a^+^/MHC-II^+^ DCs in per view was counted and statistical analysis was performed. Significant differences between the treated groups and the control groups are identified as. Scale bar = 100 μm, *n* = 10 *p < 0.05

DCs are the most potent antigen-presenting cells that bridge innate and adaptive immunity in vivo [3, 4]. The study used IF analysis via dual staining with antibody specific to the DCs markers to detect DCs. SWC3a positive cells were stained red, MHC II positive cells were stained green, double positive cells were SWC3a^+^ /MHC-II^+^ DCs, which were stained yellow. Our results showed that the number of SWC3a^+^/MHC-II^+^ DCs significantly increased in ileum after oral inactivated PEDV (Fig. 4).

### The number of ileum IgA^+^ cells increases after oral inactivated PEDV

**Fig. 5:**
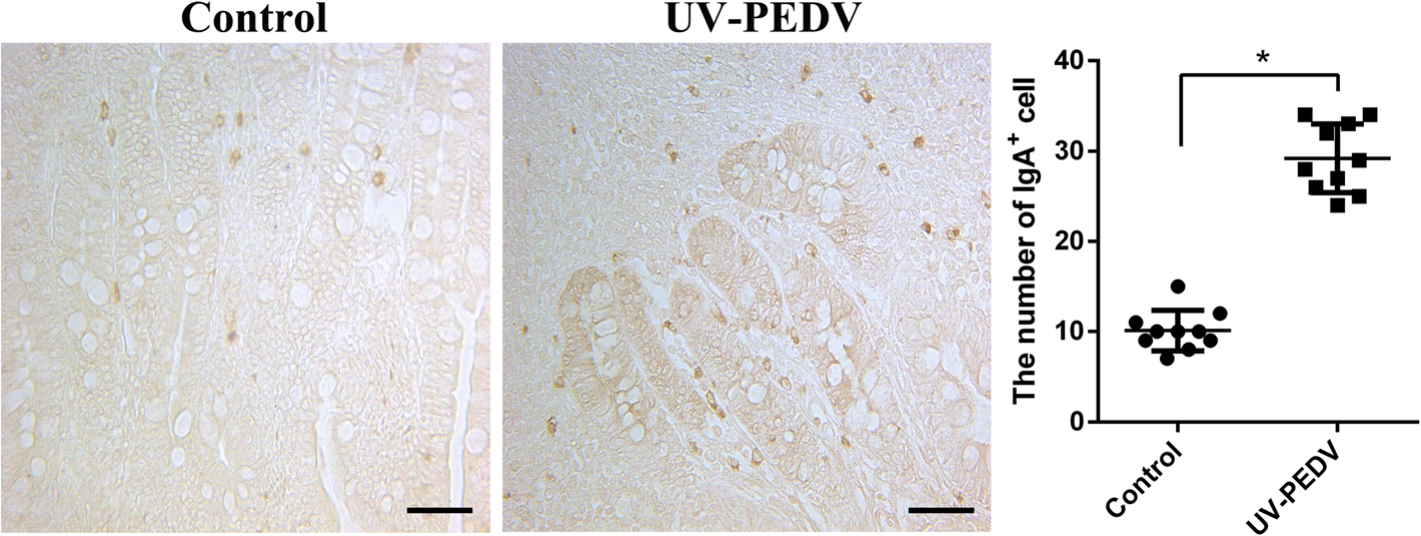
The number of ileum IgA^+^ cells increases after oral inactivated PEDV. IgA^+^ cells were showed by immumohistochemical staining. The number of IgA^+^ cells in per view was counted and statistical analysis was performed. Scale bar = 100 *μ*m, n = 10, * *P* < 0.05, ** *P* < 0.01

IgA favors both maintenance of non-invasive commensal bacteria and neutralization of invasive pathogens [16]. Besides neutralizing pathogens in the intestinal lumen, IgA can intercept microbes and toxins inside intestinal epithelial cells [17]. The distribution patterns of IgA^+^ cells in ileum were examined by IHC. The positive cells were stained brown. The IgA^+^ positive cells represented the presence of SIgA molecular adhesion. The number of ileum IgA+ cells have significantly different between oral inoculation of inactivated PEDV group and control group (Fig. 5).

### The levels of anti-PEDV specific IgG antibody in the serum and SIgA antibody in the feces increases after oral inactivated PEDV

**Fig. 6:**
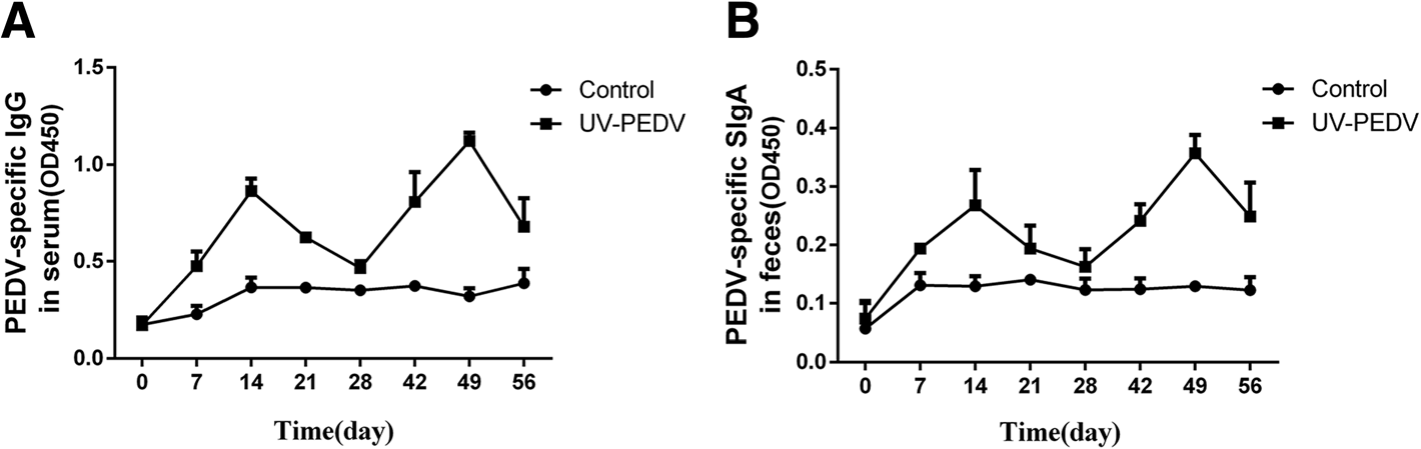
The levels of anti-PEDV specific IgG antibody in the serum and SIgA antibody in the feces increases after oral inoculation of inactivated PEDV. The levels of anti-PEDV specific IgG antibody in the serum (**a**) and SIgA antibody in the feces (**b**) was determined by ELISA. All data are shown as the mean ± S.D. *P < 0.05; **P < 0.01

The levels of anti-PEDV specific IgG antibody in the serum and SIgA antibody in the feces was determined by ELISA. As shown in the Fig. 6, the levels of anti-PEDV specific IgG antibody in the serum increased after oral immunization with UV-inactivated PEDV (*p* < 0.01) as compared to those in the control groups of piglets orally immunized with PBS after the first vaccinations. At 14 and 49 day, anti-PEDV specific IgG antibody in the serum reached a relatively higher level (*p* < 0.01) (Fig. 6a). Similar variation tendency of SIgA titers was detected in the feces (Fig. 6b). No significant difference was detected in the negative control group during the experiment.

## Discussion

The small intestinal mucosal immune system is the first line of defense against a wide variety of exogenous molecules [2]. Peyer’s Patches (PPs) was named after their detailed description by the Swiss pathologist Johann Conrad Peyer in 1677 [18]. PPs play an important role in distinguishing between potentially harmful agents and common food ingredients within the ingesta as a key component of the gut-associated lymphoid tissue [2, 19]. PPs serve as the primary inductive sites for intestinal immunity [19].

Van Kruiningen et al.noted that the PPs occupied most of the area in the ileum [20, 21]. Within PPs, DCs in the dome and interfollicular areas can uptake antigens from the gut lumen by extending dendrites into epithelium, then and process and present to lymphocytes, which effectively antigen produces an immune response [4, 14, 15]. DCs derived from blood and bone marrow precursors can’t represent the real immune status of the body. Mice are the most commonly used animal models, but because of their small size they have a low amount of DCs in their intestinal mucosal. Moreover, the immune system of mice have less than 10% similarity to humans [22]. In addition, it is difficult to identify and collect DCs from human PPs, study of DCs from human PPs is difficult to achieve [2]. Therefore, the current study is about the isolation of DCs in porcine PPs. Pigs are very similar in anatomy, physiology and genetics to humans. Moreover, the immune system of pigs is over 80% similar to humans [22–24]. A large number of experiments have proved that pigs can be used as an ideal experimental animal model for the establishment of human disease infection and can be used to isolate and characterize the DCs in porcine PPs [25–27]. So, it is important to establish a method to isolate a number of functional DCs in vitro. In this study, we establish a method to isolate DCs in porcine PPs, which could contribute to further study on the role of DCs in porcine PPs.

PEDV infected the host in the mucosal surface of the intestine. Therefore, it is important to develop oral mucosa vaccines that can elicit effective mucosal immune responses against PEDV infection. An oral vaccination is an efficacious strategy owing to induction of potent humoural and mucosal immune responses and it offers significant advantages such as easy delivery, labor health safety in comparison to other vaccination routes such as intramuscular. Currently live attenuated PEDV vaccines are commercially used [28]. Inactivated whole-virus vaccines and live PEDV can induce significantly protective immune responses respectively. But the problem occurs when live attenuated PEDV mutates into virulent strain or the small intestine mucosal immunity is insufficient, the virus proliferates in the small intestine and can be easily spread in the piglets and cause PED. However, inactivated whole-virus vaccines can solve the secure problem and be effective as well.

PPs is the main site of induction of IgA-producing plasma B cells by DCs loaded with commensal bacteria [29]. Inactivated viruses are usually not sufficiently effective in eliciting local mucosal immune response. DCs play a significant role in the application of inactivated PEDV oral vaccine. After oral administration of antigens, PPs are the first places of T-cell-specific priming and proliferation in the gut. DCs promote lymphocytes proliferation and secreting cytokines against an array of invading pathogens including PEDV. DCs derived from spleen (SP) exhibit strong functional differences as compared to DCs derived from PPs [30]. DCs from PPs are more potent in stimulating allogeneic T-cells proliferation compared with DCs from SP. As well as, the DCs derived from PPs are able to prime the production of IL-4 and IL-10 (Th2 anti-inflammatory cytokines), while DCs from SP lack this ability [30]. Therefore, designing vaccines targeting DCs may be potential agents for the suppression of viral infections. In this study, our results indicated that DCs in porcine PPs after oral administration of inactivated PEDV not only can stimulate the proliferation of allogeneic lymphocytes, but also can stimulate lymphocytes to secrete cytokines (IL-1 and IL-4), after the mixed reaction of DCs and allogeneic lymphocytes. In addition, we also found that the number of ileum IgA^+^ cells and DCs in porcine ileum significantly increased in piglets in vitro and the levels of anti-PEDV specific IgG antibody in the serum and SIgA antibody in the feces increases after oral administration of inactivated PEDV.

## Conclusion

For the first time we investigated the role of DCs in porcine PPs after oral inactivated PEDV. Our findings indicate that oral administration of inactivated PEDV activate DCs in porcine Peyer’s patches and inactivated PEDV may be a useful and safe vaccine to trigger adaptive immunity.

## Abbreviations

APC: Antigen-presenting cells
cDCs: Conventional DCs
DCs: Dendritic cells
FACS: Fluorescence activated cell sorting
GALT: Gut associated lymphoid tissue
Mo-DCs: Monocyte-derived dendritic cells
pDCs: Plasmacytoid DCs
PED: porcine epidemic diarrhea
PEDV: Porcine epidemic diarrhea virus
PPs: Peyer’s patches
SABC: Strept Avidin-Biotin Complex
UV-PEDV: Ultraviolet rays inactivated PEDV. HBSS Hank’s Balanced Salt Solution
