# Correction to: Oral administration of inactivated porcine epidemic diarrhea virus activate DCs in porcine Peyer’s patches

**DOI:** 10.1186/s12917-018-1631-9

**Published:** 2018-10-03

**Authors:** Chen Yuan, En Zhang, Lulu Huang, Jialu Wang, Qian Yang

**Affiliations:** MOE Joint International Research Laboratory of Animal Health and Food Safety, College of veterinary medicine, Nanjing Agricultural University, Weigang 1, Nanjing, Jiangsu 210095 People’s Republic of China

## Correction

The original article [1] contained an error whereby the leftmost graph in Fig. 1a mistakenly had its x-axis labelled as ‘CFST’; this has now been corrected to ‘CFSE’.
